# Functional variants of *TIM-3/HAVCR2* 3′UTR in lymphoblastoid cell lines

**DOI:** 10.4155/fsoa-2017-0121

**Published:** 2018-03-15

**Authors:** Feifei Pu, Fengxia Chen, Zhicai Zhang, Jing Feng, Ping Xia

**Affiliations:** 1 Department of Orthopedics, Wuhan No.1 Hospital, Wuhan Integrated TCM & Western Medicine Hospital, Wuhan, Hubei 430022, PR China; 2 Department of Medical Oncology, General Hospital of The Yangtze River Shipping, Wuhan, Hubei 430022, PR China; 3 Department of Orthopedics, Union Hospital, Tongji Medical College, Huazhong University of Science & Technology, Wuhan, Hubei 430022, PR China

**Keywords:** genetic, miRNA, polymorphism, *TIM-3/HAVCR2*, variant

## Abstract

**Aim:**

Variants of *TIM-3/HAVCR2* 3′UTR miRNA binding sites are significantly associated with cancer; however, roles in post-transcriptional regulation have not been elucidated.

**Methods:**

The regulatory and coding region single nucleotide polymorphisms (SNPs) of *TIM-3/HAVCR2* were identified using an online database. Single nucleotide polymorphism Function Prediction was used to predict potential functional relevance of miRNA binding sites.

**Results:**

The analysis indicated rs9313439, rs4704846, rs3087616 and rs1036199 affect possible miRNA binding sites in *TIM-3/HAVCR2* 3′UTR. We used additional data on genotypes and limited minor allele frequency >5% in the HapMap populations. Only rs3087616 and rs4704846 were significantly associated with *TIM-3/HAVCR2*.

**Conclusion:**

Both rs3087616 and rs4704846 could be putative variants mediating post-transcriptional regulation of the *TIM-3/HAVCR2*. Deeper understanding of how 3′UTR variants influence the activity by *TIM-3/HAVCR2* for therapy against cancer.

## Lay abstract

*TIM-3/HAVCR2* is involved in the pathogenesis of malignant tumors and progression of various types of cancer. Deeper understanding of how 3′UTR variants influence activity by *TIM-3/HAVCR2* is useful for research into cancer therapy. In this study, single nucleotide polymorphism Function Prediction was used to predict potential functional relevance of miRNA binding sites. We found rs3087616 and rs4704846 were of interest. Our findings indicate that the 3′UTR variants regulate the activity of *TIM-3/HAVCR2* and could aid in targeting the *TIM-3/HAVCR2* pathway for cancer treatment.

Cancer is a common, potentially fatal disease resulting from complex interactions between environmental and genetic factors [1]. Studies are increasingly focused on the role of gene polymorphisms in the etiology of cancers. Growing evidence indicates that single nucleotide polymorphisms (SNPs) play an important role in carcinogenesis; gene polymorphisms have been implicated in response to methotrexate toxicity in high-grade osteosarcoma [2,3]. Cancer immunotherapy has produced impressive clinical results in recent years. The TIM-3 mediates immune tolerance in tumor immunity. Thus, targeting TIM-3 has emerged as a promising approach for enhancement of current immunotherapy.

The *TIM-3/HAVCR2* gene is located in the chromosomal region 5q33.3 in humans [4]. Despite a large amount of experimental data showing an immunosuppressive function of TIM-3 *in vivo*, the exact mechanisms of this action are not well understood. The genetic variations of *TIM-3/HAVCR2* may contribute to the development of cancers [5,6]. Overexpression of *TIM-3/HAVCR2* is associated with a poor prognosis in squamous cell carcinoma, and in colorectal, gastric and breast cancers [7–10]. Additionally, *TIM-3/HAVCR2* is intimately involved in the pathogenesis of malignant tumors and progression of various types of cancer.

It is well known that variants of miRNA binding sites can alter gene function. miRNAs can regulate the activity of *TIM-3/HAVCR2*, and dysregulation of miRNA is implicated in neoplasms. Fooladinezhad and colleagues report that TIM-3 is a specific surface marker for leukemic stem cells and is highly expressed on leukemic stem cells compared with normal bone marrow cells and hematopoietic stem cells. Studies indicate that miRNAs can affect the progression of acute myeloma leukemia by targeting the expression of different genes, such as that of *TIM-3*. Bioinformatics assessments indicate that miR-330-5p may robustly inhibit the expression of TIM-3, and miR-330-5p has been shown to have a strong inhibitory effect on the expression of TIM-3 in an acute myeloma leukemia cell line [11]. In this study, we performed a bioinformatics analysis, using the HapMap database, to examine whether the *TIM-3/HAVCR2* 3′UTR variants are associated with tumorigenesis and tumor progression.

## Materials & methods

The SNPs of *TIM-3/HAVCR2* were identified, in the regulatory and coding regions, using an online database [12]. The bioinformatics tool SNP Function Prediction (FuncPred; [13]) was used to predict the potential functional relevance of the miRNA binding sites. Additionally, we limited our analysis to SNPs with minor allele frequency (MAF) >5% in the HapMap populations, ‘Utah residents with Northern and western European ancestry (CEU)’ and calculated pairwise linkage disequilibrium (LD) values of all the SNPs in the same gene. Then, we selected SNPs that were not in LD (r^2^ <0.8) and constructed the LD maps of *TIM-3/HAVCR2* SNPs using a web service [13].

## Results

### Selected variants of the *TIM-3/HAVCR2* 3′UTR & putative miRNA binding sites

In total, 43 SNPs were identified in the *TIM-3/HAVCR2* 3′UTR; only rs3087616 and rs4704846 have a known MAF value >0.05. According to the bioinformatics analysis, these two SNPs were predicted to affect the activity of the miRNA binding site, as shown in Table 1. The predicted functions of the *TIM-3/HAVCR2* 3′UTR SNPs, in different ethnic groups are shown in Table 2. The comparisons of LD in common SNPs, among the different populations are listed in Table 3. The most extensively studied SNPs affect the putative binding sites of miRNAs [13].

**Table 1.** **Selected single nucleotide polymorphisms of *TIM-3/HAVCR2* 3′UTR and putative miRNA binding sites.**

**Name**	**Alleles**	**MAF**	**Putative miRNA binding sites**
rs3087616	C/T	0.1116	hsa-miR-1250, hsa-miR-1827, hsa-miR-196a, hsa-miR-196b, hsa-miR-34a, hsa-miR-34c-5p, hsa-miR-449a, hsa-miR-449b and hsa-miR-582-5p

rs4704846	A/G	0.1581	hsa-miR-18b, hsa-miR-379, hsa-miR-554, hsa-miR-631, hsa-let-7a, hsa-let-7e, hsa-let-7f, hsa-let-7g and hsa-let-7i

3′UTR: 3′ Untranslated region; MAF: Minor allele frequency; NA: Not available.

**Table 2.** **Single nucleotide polymorphisms function prediction results of *TIM-3/HAVCR2* 3′UTR in different races.**

**Name**	**Alleles**	**African**	**Asian**	**European**	**African–American**	**Hispanics**
rs3087616	C/T	0.822	0.991	0.804	0.833	NA

rs4704846	A/G	0.685	0.989	0.816	NA	NA

3′UTR: 3′ Untranslated region; NA: Not available.

**Table 3.** **Comparison of linkage disequilibrium of common single nucleotide polymorphisms among the populations.**

**Name**	**Alleles**	**CEU**	**CHB**	**CHD**	**GIH**	**LWK**	**MEX**	**YRI**
rs3087616	C/T	0.844	0.988	0.994	0.938	0.817	0.92	0.836

rs4704846	A/G	0.816	0.989	NA	NA	NA	NA	0.704

NA: Not available.

### Calculating the LD of all SNPs in the *TIM-3/HAVCR2* gene

**Figure 1.**
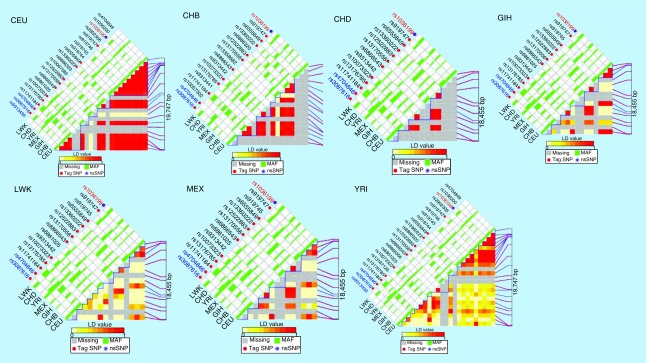
**A linkage disequilibrium plot of the *TIM-3/HAVCR2* region generated using the single nucleotide polymorphism Function Prediction software (FuncPred).** Each square number represents the pairwise r^2^ correlations between the two relevant SNPs. The color of each SNP spot reflects its D′ value, which changes from red to white as the D′ value decreases. MAF: Minor allele frequency; SNP: Single nucleotide polymorphism.

The bioinformatics tool FuncPred [14] was used to identify the potential functional relevance of the SNPs. To select the SNPs that were not in LD (r^2^ <0.8), we calculated the pairwise LD values of all SNPs in the same gene and plotted the LD maps of these *TIM-3/HAVCR2* SNPs using FuncPred. The pairwise r^2^ correlations between the two relevant SNPs were represented by each square number. The color of each SNP spot reflects its D′ value; when the D′ value decreases, the color changes from red to white. The haplotype blocks were estimated using the FuncPred software. The MAF of all the above alleles was greater than 0.05. In our study, the polymorphisms, rs3087616 and rs4704846 were the predicted tag SNPs, as indicated by the LD plot of the *TIM-3/HAVCR2* region generated using FuncPred. Each square number represents the pairwise r^2^ correlations between the two relevant SNPs. The color of each SNP spot reflects its D′ value, which changes from red to white as the D′ value decreases. An SNP is shown in Figure 1.

## Discussion

Numerous studies, such as multiple tissue gene expression, animal models and clinical trials suggest that *TIM-3/HAVCR2* plays an important role in carcinogenesis. Various groups have analyzed the link between *TIM-3/HAVCR2* polymorphisms and the risk of cancer [7–10]. An increased level of TIM-3 is correlated with poor survival in patients with cancer. The soluble form of TIM-3 has been shown to reduce the antigen-specific T-cell response and downregulate the antitumor activity *in vivo* [15]. Another study revealed that blockade of TIM-3 can reverse the impaired phenotype of natural killer cells in patients with metastatic melanoma [16], highlighting the potential of TIM-3-targeted therapy. Nevertheless, despite all the promising data shown in preclinical models, the role of TIM-3 has not been evaluated in clinical trials, possibly because of insufficient evidence on the role of TIM-3 in clinical patients with cancer.

Many studies confirm that SNPs, in cancer-related genes, can contribute to individual susceptibility to cancer by affecting gene expression and function [17,18]. In recent study, the results have confirmed the bioinformatics prediction of suppressive effect of miR-125a-3p on TIM-3 with miRWalk and TargetScan softwares [19].  Our study supports the notion that mutations in miRNA binding regions contribute to altered gene function. Although our findings indicate that rs9313439, rs4704846, rs3087616 and rs1036199, in the *TIM-3/HAVCR2* 3′UTR, affect the potential miRNA binding sites, we found that rs3087616 and rs4704846 were significantly associated with *TIM-3/HAVCR2* in the lymphoblastoid cell lines. The polymorphisms rs3087616 and rs4704846 were the predicted tag SNPs in our study, suggesting that these variants may contribute to the *TIM-3/HAVCR2* post-transcriptional regulation. Our results indicate a mechanism, underlying the roles of rs3087616 and rs4704846, in the regulation of *TIM-3/HAVCR2* expression, and provide an explanation for the susceptibility to tumor formation associated with these SNPs. It is possible that the genetic variants in the *TIM-3/HAVCR2* 3′UTR modulate its expression, and the variants affecting the *TIM-3/HAVCR2* miRNA binding sites are associated with carcinogenesis.

## Conclusion & future perspective

Our results suggest that the SNPs, rs3087616 and rs4704846 of *TIM-3/HAVCR2*, involved in the pathways of immunoblastic escape, can be used as predictive factors for the clinical outcome in anticancer therapy. Our study contributes to the understanding of how the miRNA variants of the *TIM-3/HAVCR2*3′UTR may regulate the expression of mRNA. Translation of pharmacogenetic predictors into clinical practice may lead to an improved planning and outcome of cancer treatment. In addition, further functional analysis is necessary to validate the promoter CpG islands and SNPs in the 3′UTR to allow for investigation of *TIM-3/HAVCR2* gene regulation as a potential therapy for cancer.

*TIM-3/HAVCR2* variants influence post-transcriptional regulation. miRNA-mediated regulation is important in cancer-associated gene expression, particularly with respect to the development of prognostic and diagnostic markers of malignancy. The 3′UTR variants could regulate the activity of *TIM-3/HAVCR2* and may aid in targeting the *TIM-3/HAVCR2* pathway for cancer treatment. However, the mechanism underlying the association of *TIM-3/HAVCR2* transcriptional activity with the variants in the 3′UTR requires validation by functional analysis.

**Executive summary**

- The single nucleotide polymorphisms (SNPs) of *TIM-3/HAVCR2* were identified by using an online database.
- The bioinformatics tool SNP Function Prediction was used to predict the potential functional relevance of the miRNA binding sites.
- Selected variants of the *TIM-3/HAVCR2* 3′UTR and putative miRNA binding sites.
- Calculated the linkage disequilibrium of all SNPs in the *TIM-3/HAVCR2* gene.
- Our study contributes to the understanding of how the miRNA variants of the *TIM-3/HAVCR2* 3′UTR may regulate the expression of mRNA.
